# The effects of online tourism information quality on conative destination image: The mediating role of resonance

**DOI:** 10.3389/fpsyg.2023.1140519

**Published:** 2023-03-02

**Authors:** Xueyi Wang, Xin Wang, Ivan Ka Wai Lai

**Affiliations:** ^1^Faculty of International Tourism and Management, City University of Macau, Macao, Macao SAR, China; ^2^Research Center for Culture and Tourism Law, Hangzhou City University, Hangzhou, Zhejiang, China

**Keywords:** online tourism information quality, cognitive resonance, emotional resonance, conative destination image, destination marketing strategies

## Abstract

With the increasing popularity of mobile applications, people enjoy browsing online tourism information on social media. This information may cause psychological resonance, which in turn stimulates travel intentions. This study examined the relationship between online travel information quality (OTIQ), resonance, and conative destination image. A partial least squares structural equation model was used to analyze the survey data of 426 users who recently used social media to browse online tourism information. The results show that four dimensions of OTIQ (value-added, relevancy, completeness, and design) affect cognitive resonance, and three dimensions of OTIQ (interestingness, design, and amount of information) affect emotional resonance. Both cognitive resonance and emotional resonance directly affect the conative destination image. This study contributes to online tourism marketing research by identifying the factors of OTIQ that rise tourists’ resonance. It also contributes to destination image research by extending the application of resonance theory and examining the role of cognitive resonance and emotional resonance in forming a conative destination image. Understanding how QTIQ builds a destination image can help destinations improve the quality of online tourism information to attract potential tourists. This study also provides recommendations to destination marketers to formulate appropriate marketing strategies in the age of innovative technology.

## 1. Introduction

Social media as a new marketing tool for tourism destinations, its mechanism of influence of online tourism information on destination image formation has been receiving attention from the tourism industry and academia (Lam et al., 2020). By July 2022, the number of social media users worldwide had surpassed 4.7 billion, and they used social media for more than 2 h a day on average (Kepios, 2022). The valuable information provided by online social media not only makes new marketing of commodities, services, and communication possible but also changes the way people make travel decisions (Khan, 2017). According to a U.S. statistic, 97% of millennials post photos on social media while on vacation. And about 52% of travelers decide to go to a specific destination after seeing photos/videos of friends, family, or peers on social media (Statistics, 2022). Every week, over 1 million travel-related hashtags are searched on Instagram (a worldwide popular social media app; Statistics, 2022). Social media has become their first-choice channel for getting tourism information (Wang et al., 2022). Social media users watch travel pictures, opinions, reviews, comments, and travel experiences shared by others on social media, either automatically pushed by Apps or by their deliberated search (Majeed et al., 2020). Although we know that a series of tourist information generated by users has shaped the image of a destination in people’s minds, which influences people’s travel decision-making (Fan et al., 2020), we do not know what types of information influence people most in choosing a destination. Therefore, a study is needed to understand the various tourism information on social media and its impact on online tourism marketing.

Recent studies indicated that online tourism information quality (OTIQ) is an important indicator in shaping the online tourism market and contributing to the formation of destination image (Kim et al., 2017). Researchers have examined the effects of influential online tourism information on online users’ travel purchases intention (Hateftabar, 2022), visit intentions (Cheng et al., 2020), and word-of-mouth recommendations (Majeed et al., 2020), which are the components of destination conative image. Although previous studies have indicated that online information content is the key factor influencing tourists’ conative behavior (Lam et al., 2020; Majeed et al., 2020), there is a lack of studies to explain the mechanism of how OTIQ influences tourists’ conative behaviors regarding the formation of the conative destination image.

The resonance theory originates from sociological research and emphasizes positive cognitive and emotional consequences (Giorgi, 2017). Researchers applied the resonance theory to explain how customers can match the information with their internal worldview when they are receiving online information (Camilleri and Kozak, 2022). The resonance theory states that adequate information will arouse the audience’s cognitive resonance and emotional resonance, and they can influence customers’ further behavioral intentions (Cheng et al., 2020). A literature search of mainstream databases (EBSCOhost, Scopus, and ScienceDirect) revealed that there were only two empirical tourism studies on resonance. These two studies tested the effect of users’ resonance with travel blogs on word-of-mouth and travel behavioral intentions (Cheng et al., 2020; Mohanty et al., 2022). In this case, it is possible that high-quality online information can rise tourists’ resonance reactions, ultimately influencing tourists’ perceptions of destination conative image. However, no research has been found on the psychological resonance of receiving high-quality tourism information in tourism research.

This study aims to examine the relationship between online tourism information quality (OTIQ), resonance, and conative destination image. Firstly, this study contributes to online tourism marketing research by identifying the factors of OTIQ that rise tourists’ resonance, thereby prompting them to generate a conative destination image. Understanding this mechanism helps destinations improve the quality of online tourism information to attract potential social media users to become destination tourists. Secondly, this study extends the application of resonance theory in destination image research by examining the role of cognitive resonance and emotional resonance in forming a conative destination image. Finally, the results of this study help destination marketers grasp accurate online tourism information and effectively carry out online tourism marketing promotion.

The study is organized as follows. A brief literature review of online tourism information quality, resonance theory, and conative destination image is provided in the next section. Then, the research methods and data analysis are described in Sections “Research hypotheses” and “Research methods”, respectively. In Section “Findings”, conclusions are drawn, the theoretical and practical implications are discussed, and the limitations of this study and recommendations for further research are presented.

## 2. Literature review

### 2.1. Conative destination image

Previous studies on the destination image have shown that destination image can affect a destination’s competitiveness in the market (Liang and Lai, 2022), thus how a destination can stand out from a favorable destination image is one of the most explored areas in tourism research. Hunt (1975) defined destination image as an individual’s beliefs, impressions, and perceptions of a specific place. Initially, researchers proposed a two-dimensional tourism destination image model, in which the tourism destination image contains both a cognitive image and an affective image (Gartner, 1994). The cognitive image relates to a person’s knowledge and beliefs about a tourism destination, and the affective image relates to how they feel about the destination (Baloglu and McCleary, 1999). Later on, researchers developed a conative destination image construct, which involves tourists’ behavioral intentions (Ryan and Ninov, 2011). A conative destination image is a collection of future actions, and it contains three test items, i.e., “intention to recommend, positive word of mouth, intention to revisit” (Woosnam et al., 2020). Lojo et al. (2020) stated that to effectively market a destination and form a market position, it is most important to identify the factors that influence the formation of the conative image of the destination. Besides cognitive image and emotional image, previous articles have shown that many factors (e.g., travel experience, positive emotions, etc.) can influence recommendation intention, word-of-mouth, and revisit intention (Hosany et al., 2017; Wang et al., 2023). However, most studies examined the factors for the conative image based on travel experience after visiting a destination. For tourists before visiting a destination, Wang et al. (2020) pointed out that their conative destination image is highly susceptible to user-generated tourism content on social media, however, there is a lack of studies to link how online tourism information affects tourists’ psychological status, and then influences tourists’ conative image.

### 2.2. Online tourism information quality

At the beginning of the 21st century, tourism information began to appear on the internet and gradually evolved into a channel for tourism organizations to communicate with tourists (Xiang and Gretzel, 2010). The emergence of Web 2.0 makes tourists easy to create and share information with other tourists on social media (Aghaei, 2012). Therefore, social media have become the most influential marketing tool for the public, businesses, and government organizations (Hays et al., 2013), because tourists consider social media to be the most trusted source of information about a destination (Fotis et al., 2012). Tourists can consult travel information on social media to support their travel decisions pre-trip, during a trip, and post-trip (Wang et al., 2022).

As information recipients, tourists obtain online travel information on social media published by information providers (Lu et al., 2016). However, tourists may be confused by the vast amount of online travel information pushed by information providers (Dharmasena and Jayathilaka, 2021), therefore, they have to pay attention to the quality of online travel information. However, previous studies mainly focused on the content of the travel information rather than the quality of travel information until Kim et al. (2017) introduced the concept of the OTIQ. Based on Chaiken’s (1980) heuristic system model, Kim et al. (2017) developed a multi-level OTIQ model for social media that includes content qualities (i.e., value-added, relevancy, timeliness, completeness, and interestingness) and non-content qualities (i.e., web page design and amount of information). Researchers have applied the multidimensional OTIQ scale to examine the impact of online travel information on the formation of the destination image in different scenarios (Rodríguez et al., 2019; Guo and Pesonen, 2022). However, these studies only tested tourists’ behavioral intentions when viewing online information on social media without investigating their psychological changes. There is a lack of studies on how online information affects travelers’ psychological status.

### 2.3. The role of resonance theory

Resonance is one of the most widespread sociological concepts that was initially used to explain an individual’s understanding of an organizational framework (Snow et al., 1986). Researchers have applied it to describe the fit between information and audience worldviews (McDonnell et al., 2017). There are two main types of resonance, cognitive resonance and emotional resonance. Cognitive resonance is based on the audience’s beliefs and understanding, and emotional resonance is based on the audience’s feelings, passions, and desires (Giorgi, 2017). These two types of resonance lead to positive consequences (Snow et al., 1986; Su et al., 2019). For their antecedents, researchers found that cognitive resonance can be achieved when people can interpret their understanding of information in a way that matches their expectations (Shang et al., 2017), and emotional resonance can be achieved when acquired information arises people’s curiosity and desire (Kang et al., 2020). Emotional resonance usually interpenetrates with cognitive resonance and eventually leads to a strong resonance that lasts for a period of time (McDonnell et al., 2017). Therefore, if tourists find that the travel information on social media meets their expectations and generates desires, they will have cognitive resonance and emotional resonance. However, what types of online travel information (content qualities and non-content qualities) can arise tourists’ cognitive resonance and emotional resonance are still a question that should be investigated.

## 3. Research hypotheses

In recent years, social media has been recognized as the most important source of acquiring tourism information (Lee et al., 2019). People are not only read travel information on social media but also involve in discussing travel information in the comment area (Camilleri and Kozak, 2022). According to the multi-level OTIQ model for social media (Kim et al., 2017), tourists would receive content qualities and non-content qualities (value-added, relevancy, timeliness, completeness, interestingness, design, and amount of information) of online information when they browse destination travel information on social media. When the information people received is consistent with their expectation and meet their demands, they are likely to have cognitive resonance with them (McDonnell et al., 2017). On the other hand, when the information people received is interesting and amazing, this information is more likely to facilitate conversations and emotional exchanges with the audience (Mangold and Faulds, 2009), therefore, they are more likely to respond emotionally to the information content (Shang et al., 2017). In general, the information posted online includes attractions and accommodations in a destination (Wang et al., 2022), which helps tourists plan their trips. Furthermore, online information about a destination also includes tourism activities and entertainment (Wong et al., 2020), which stimulates tourists’ interests. Then, if tourists find the online travel information sufficient, useful, and relevant, they will have a cognitive resonance with the online travel information; if tourists find the online travel information interesting and amazing, they will have an emotional resonance with the online travel information. Therefore, the following two sets of hypotheses are proposed.

*H1:* When tourists browse travel information on social media, its value-added (a), relevancy (b), timeliness (c), completeness (d), interestingness (e), design (f), amount of information (g), positively influences their cognitive resonance.

*H2:* When tourists browse travel information on social media, its value-added (a), relevancy (b), timeliness (c), completeness (d), interestingness (e), design (f), amount of information (g), positively influences their emotional resonance.

Cognitive resonance and emotional resonance are not mutually independent, these two resonances have a complex permeating process, as it is not only the mundane that people focus on but the emotional touch that the event brings (Giorgi, 2017). Since people searching for information is driven by emotions (McDonnell et al., 2017), tourists’ emotions will be affected by their cognitive resonance from the context of online travel information they obtained from social media. If tourists cannot get cognitive resonance from online travel information, they will not have a feeling of emotional resonance. It means that tourists find online travel information interesting once they find online travel information useful. Therefore, cognitive resonance is a necessary condition for tourists to have emotional resonance from online travel information.

*H3:* Tourists’ cognitive resonance positively influences their emotional resonance.

Giorgi (2017) suggested that resonance is an antecedent of the audiences’ intentional output. Previous studies have shown a positive relationship between engagement with travel information on social media and tourists’ behavioral intention (Tran, 2020). Mohanty et al. (2022) found that cognitive resonance and emotional resonance can facilitate social media engagement behaviors. Therefore, the cognitive resonance and emotional resonance obtained by engaged tourists from online travel information may contribute to the formation of the destination image. Cheng et al. (2020) stated that when browsing travel information on social media, tourists are emotionally inspired by the received information, which in turn forms the destination image or forms a visit intention. Wu and Lai (2022) pointed out that tourists are influenced by media information and are keen to go to destinations which impress them and conform to their self-congruity. This means that tourists’ cognitive resonance and emotional resonance to online travel information may directly influence their perception of the conative image of a destination. Therefore, the following hypotheses are proposed.

*H4:* Tourists’ (a) cognitive resonance and (b) emotional resonance positively influence their conative image of a destination.

**Figure 1:**
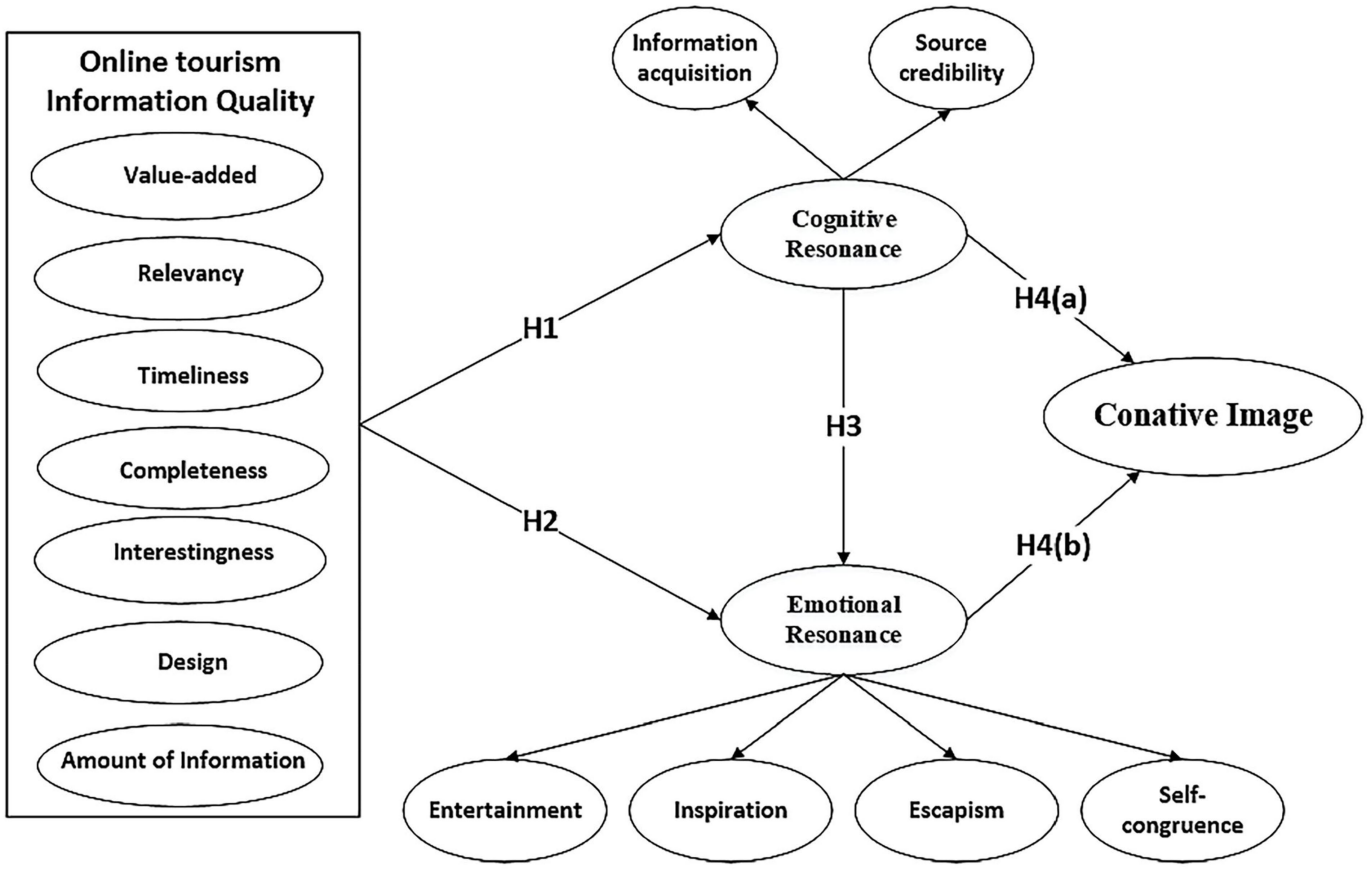
Research model.

This study aims to examine the relationship between OTIQ, cognitive resonance, emotional resonance, and conative image. The hypothetical model is shown in Figure 1.

## 4. Research methods

### 4.1. Measurement

The measurable items of seven factors (amount of information, completeness, design, interestingness, relevancy, timeliness, and value-added) of OTIQ used in this study are inspired by Kim et al. (2017). The measurable items of cognitive resonance and affective resonance are borrowed from Cheng et al. (2020). The measurable items of the conative image are borrowed from Afshardoost and Eshaghi (2020). All the measurement scales have been well-validated in previous studies. To suit the research setting, an expert meeting was conducted to slightly adjust the original measures according to the content of this case. The panel of experts includes two scholars in tourism, a social media company executive, a member of the tourism board, and three tourists who have used social media for planning travel. Experts recommended some modifications such as removing two items referring to video quality and no specific location in measuring conative image.

### 4.2. Questionnaire design and data collection

This study divided the questionnaire into three sections. The first section consisted of screening questions to ensure that respondents met the criteria for the study. Respondents were screened based on three criteria: (i) being at least 18 years old; (ii) having used social media in daily life; and (iii) having browsed tourism destination information on social media within a week. The second part consisted of seven dimensions of OTIQ, cognitive resonance and emotional resonance, and conative destination image. Respondents were asked to answer the questionnaire based on their last experience browsing online travel information. The questionnaire was measured using a 7-point Likert scale (where “1” = strongly disagree, “7” = strongly agree). The third section was about the respondents’ background information. The English questionnaire was translated into Chinese, and the Chinese questionnaire was back-translated into English by two English-Chinese translators to eliminate translation bias. Expert consultation with five tourism scholars was conducted to validate the content of the questionnaire. A pilot test was conducted with 30 social media users to further ensure the accuracy and readability of the questionnaire. Some adjustments were made to the wording of some items, such as adding explanations for specific social media platforms.

An online survey was conducted from the 1st of September to the 31st of October 2022. The online survey is a non-probability sampling method widely used in tourism research (Cho et al., 2020). Using an online survey in this study can reach more potential participants who have experience in browsing online travel experiences. 480 samples were collected. The 54 questionnaires scored the same scores on most of the questions, so only 426 samples were valid for data analysis. The effective rate of the questionnaire was 88.75%.

## 5. Findings

The partial least squares structural equation modeling (PLS-SEM) was used to evaluate the research model. Compared with CB-SEM, PLS-SEM has fewer restrictions on the normal distribution of the data (Hair et al., 2017). This study used SmartPLS (v.3.3.9) for data analysis (Ringle et al., 2015). This sample size was satisfied by a power analysis based on the part of the model with the largest number of predictors (Hair et al., 2021).

### 5.1. Sample overview

Table 1 shows the characteristics of 426 valid respondents. Among the participants, 63.1% were females. The 18–20, 21–30, and 31–40 age groups accounted for 18.8, 42.0, and 19.5%, respectively. 61.2% of participants had a bachelor’s degree. 91.8% of participants used social media 2–3 times or more a day.

**Table 1:** Respondents’ characteristics (*n* = 426).

		Frequency	Percent
Gender	Male	157	36.9
	Female	269	63.1
Age	18–20	80	18.8
	21–30	179	42.0
	31–40	83	19.5
	41–50	63	14.8
	51–60	18	4.2
	>60	3	0.7
Frequency of using social media information in daily life. (e.g., Xiaohongshu, ShakeYin, Sina Weibo, etc.).	More than 2–3 times a day	276	64.8
	2–3 times per day	115	27.0
	Less than 2–3 times a day	35	8.2
Education	Junior high school or below	54	12.6
	Associate degree/Diploma	49	11.6
	Bachelor degree	261	61.2
	Master or above	62	14.6
Monthly income (USD)	<285	154	36.2
	286–714	110	25.8
	715–1,428	90	21.1
	1,429–2,142	48	11.3
	>2,142	24	5.6

### 5.2. Reliability and validity

Table 2 shows the means, standard deviations (SD), and factor loadings for the 43 measurable items. All factor loadings were above 0.7 and ranged from 0.701–0.917. As shown in Table 3, the Cronbach’s alpha coefficients for each construct exceeded 0.7, the values of Composite Reliability and rho_A for each construct also exceeded 0.7, and the average variance extracted (AVE) scores were greater than 0.50, therefore, substantial reliability and convergent validity were achieved (Hair et al., 2019).

**Table 2:** Mean, standard deviation (SD), factor loading, and variance inflation factor (VIF) of 43 measurable items.

		Measurement item	Mean	SD	Factor loading	VIF
Online tourism information quality	Amount of information	Tourism information provided by some destination on social media is…Small in quantity…Large in quantity	4.953	1.344	0.791	1.485
		Amount of tourism information provided by some destination on social media is…Insufficient…Sufficient	5.005	1.245	0.815	1.512
		Amount of tourism information provided by some destination on social media is…Inappropriate to read… Appropriate to read	4.850	1.179	0.837	1.482
		Tourism information provided by social media (e.g., Xiaohongshu/Xinlang weibo/Douyin) is…				
	Completeness	Of sufficient depth	4.376	1.389	0.833	1.559
		Specific	4.610	1.294	0.816	1.629
		Accurate	4.310	1.337	0.854	1.717
		Interface design for tourism destination information pushed on social media is…				
	Design	Attractive	5.061	1.260	0.783	1.384
		Brand new	4.721	1.254	0.817	1.420
		Trendy	4.890	1.320	0.793	1.391
		Tourism information content provided by some destination on social media is…				
	Interestingness	Funny	4.829	1.345	0.855	1.959
		Attractive	5.146	1.338	0.853	1.779
		Interesting	4.883	1.360	0.889	2.196
	Relevancy	Relevant to my travel	5.005	1.389	0.883	2.038
		Relevant to my intention to follow some destinations on social media	4.913	1.356	0.870	2.160
		In accordance with my purpose to travel	4.678	1.375	0.865	1.898
	Timeliness	Quite new	4.819	1.305	0.788	1.410
		Continuously updated	5.068	1.312	0.832	1.488
		Quickly provided necessary information for the trip	4.878	1.490	0.798	1.435
	Value-added	Effective for planning a trip	4.812	1.292	0.865	1.975
		Useful for planning a trip	5.016	1.312	0.881	2.125
		Helpful for planning a trip	5.171	1.320	0.852	1.777
Resonance	Cognitive resonance	I got travel information from social media that interested me.	5.192	1.360	0.899	1.450
		I found out something new about this destination that I did not know before.	5.465	1.286	0.865	1.450
		Social media provides very knowledgeable information on this topic.	4.763	1.255	0.793	1.690
		Social media provides trustworthy information on this topic.	4.357	1.349	0.853	2.121
		Social media provides credible information on this topic.	4.479	1.299	0.825	1.890
		Social media provides information on this topic that is authentic.	4.441	1.342	0.833	1.970
	Emotional resonance	Social media information about the destination travel helped me to relax.	4.951	1.282	0.917	1.812
		Social media tourism information about destination made me feel entertained.	5.085	1.249	0.910	1.812
		It represented the destination in an appealing way.	5.174	1.287	0.874	1.979
		It helped me to be imaginative about the destination.	5.216	1.298	0.887	2.199
		It inspired me to visit the destination.	5.235	1.317	0.866	2.021
		I felt like I was in that place even though I did not travel in person.	4.580	1.533	0.846	2.204
		The watching experience let me imagine being there.	4.617	1.516	0.885	2.607
		I escaped from reality.	4.031	1.713	0.701	1.451
		I felt as if I was part of the traveling journey.	4.500	1.516	0.847	2.064
		The online image of social media was consistent with how I see myself	4.369	1.380	0.830	1.907
		The online image of social media was consistent with how I like to see myself.	4.620	1.343	0.795	1.737
		The online image of social media was consistent with how I believe others see me.	4.448	1.312	0.880	2.487
		The online image of social media was consistent with how I would like others to see me.	4.636	1.270	0.845	2.111
Conative Image		I have an intention to visit this destination.	5.124	1.396	0.846	1.744
		I have a willingness to speak positively about this destination.	4.960	1.449	0.858	1.830
		I have an intention to recommend this destination.	4.847	1.483	0.848	1.723

**Table 3:** Reliability, validity, and Fornell-Larcker criterion.

	Cronbach’s Alpha	rho_A	CR	AVE	Fornell-Larcker criterion													
					En	E	IA	IN	Scon	Scre	OTIA	OTIC	CI	OTID	OTII	OTIR	OTIT	OTIV
En	0.802	0.803	0.910	0.835	0.914													
E	0.839	0.854	0.893	0.677	0.531	0.823												
IA	0.715	0.724	0.875	0.778	0.524	0.264	0.882											
IN	0.848	0.849	0.908	0.767	0.701	0.545	0.531	0.876										
Scon	0.858	0.860	0.904	0.702	0.514	0.722	0.338	0.534	0.838									
Scre	0.845	0.845	0.896	0.683	0.453	0.510	0.432	0.417	0.610	0.827								
OTIA	0.748	0.756	0.855	0.664	0.558	0.423	0.488	0.577	0.487	0.436	0.815							
OTIC	0.782	0.786	0.873	0.696	0.449	0.489	0.334	0.410	0.551	0.609	0.525	0.834						
CI	0.809	0.809	0.887	0.724	0.525	0.479	0.529	0.516	0.532	0.572	0.404	0.444	0.851					
OTID	0.715	0.717	0.840	0.636	0.589	0.503	0.467	0.583	0.580	0.529	0.629	0.575	0.496	0.798				
OTII	0.833	0.836	0.900	0.750	0.578	0.413	0.499	0.604	0.533	0.470	0.557	0.586	0.486	0.638	0.866			
OTIR	0.844	0.850	0.905	0.761	0.403	0.330	0.420	0.349	0.421	0.509	0.430	0.530	0.429	0.502	0.493	0.873		
OTIT	0.731	0.734	0.848	0.650	0.486	0.394	0.405	0.470	0.419	0.467	0.575	0.638	0.391	0.618	0.527	0.598	0.806	
OTIV	0.833	0.833	0.900	0.750	0.553	0.396	0.535	0.558	0.503	0.545	0.630	0.651	0.507	0.619	0.643	0.600	0.608	0.866

CR, Composite Reliability; AVE, Average Variance Extracted; En, Entertainment; E, Escapism; IA, Information acquisition; IN, Inspiration; Scon, Self- congruence; Scre, Source credibility; OTIA, Amount of Information; OTIC, Completeness; CI, Conative image; OTID, Design; OTII, Interestingness; OTIR, Relevancy; OTIT, Timeliness; OTIV, Value-added.

The correlation between constructs was less than the square root of the AVE score (numbers on the diagonal) and all values of the heterotrait-monotrait (HTMT) ratio were below the indicator 0.9 (Henseler et al., 2015). Table 4 shows the HTMT ratios. Because of high HTMT ratios for the two pairs of ‘Design’ and ‘Amount of information’ (0.857), ‘Timeliness’ and ‘Design’ (0.855), a bootstrapping method was used to assess the inference of HTMT. The values of the confidence interval (97.5%) for both pairs are less than 1, thus, it did not violate the hypothesis of discriminant validity, and the discriminant validity was confirmed (Hair et al., 2021).

**Table 4:** Heterogeneity-Monogeneity (HTMT) ratio.

	En	E	IA	IN	Scon	Scre	OTIA	OTIC	CI	OTID	OTII	OTIR	OTIT	OTIV
En														
E	0.639													
IA	0.690	0.331												
IN	0.849	0.633	0.682											
Scon	0.618	0.850	0.425	0.627										
Scre	0.550	0.612	0.552	0.493	0.715									
OTIA	0.715	0.526	0.667	0.726	0.602	0.540								
OTIC	0.569	0.598	0.441	0.504	0.671	0.742	0.683							
CI	0.652	0.581	0.692	0.624	0.638	0.691	0.516	0.557						
OTID	0.777	0.647	0.656	0.750	0.737	0.679	0.857	0.765	0.654					
OTII	0.704	0.489	0.640	0.716	0.630	0.560	0.701	0.729	0.588	0.827				
OTIR	0.486	0.397	0.532	0.410	0.493	0.599	0.532	0.645	0.514	0.642	0.586			
OTIT	0.633	0.505	0.557	0.591	0.530	0.594	0.775	0.847	0.509	0.855	0.672	0.760		
OTIV	0.677	0.467	0.687	0.663	0.595	0.650	0.796	0.810	0.617	0.804	0.772	0.714	0.778	

En, Entertainment; E, Escapism; IA, Information acquisition; IN, Inspiration; Scon, Self- congruence; Scre, Source credibility; OTIA, Amount of Information; OTIC, Completeness; CI, Conative image; OTID, Design; OTII, Interestingness; OTIR, Relevancy; OTIT, Timeliness; OTIV, Value-added.

This study used Harman’s single-factor test to clarify the absence of common method variance. The results indicated that the first factor explained 39.079% of the variance; as such, the issue of common method variance did not exist in this study (Podsakoff et al., 2003). In addition, the values of all variance inflation factors (VIF) were below 3 (as shown in Table 2), indicating that there was no collinearity problem in this study (Hair et al., 2019).

### 5.3. Results of partial least squares analysis

**Figure 2:**
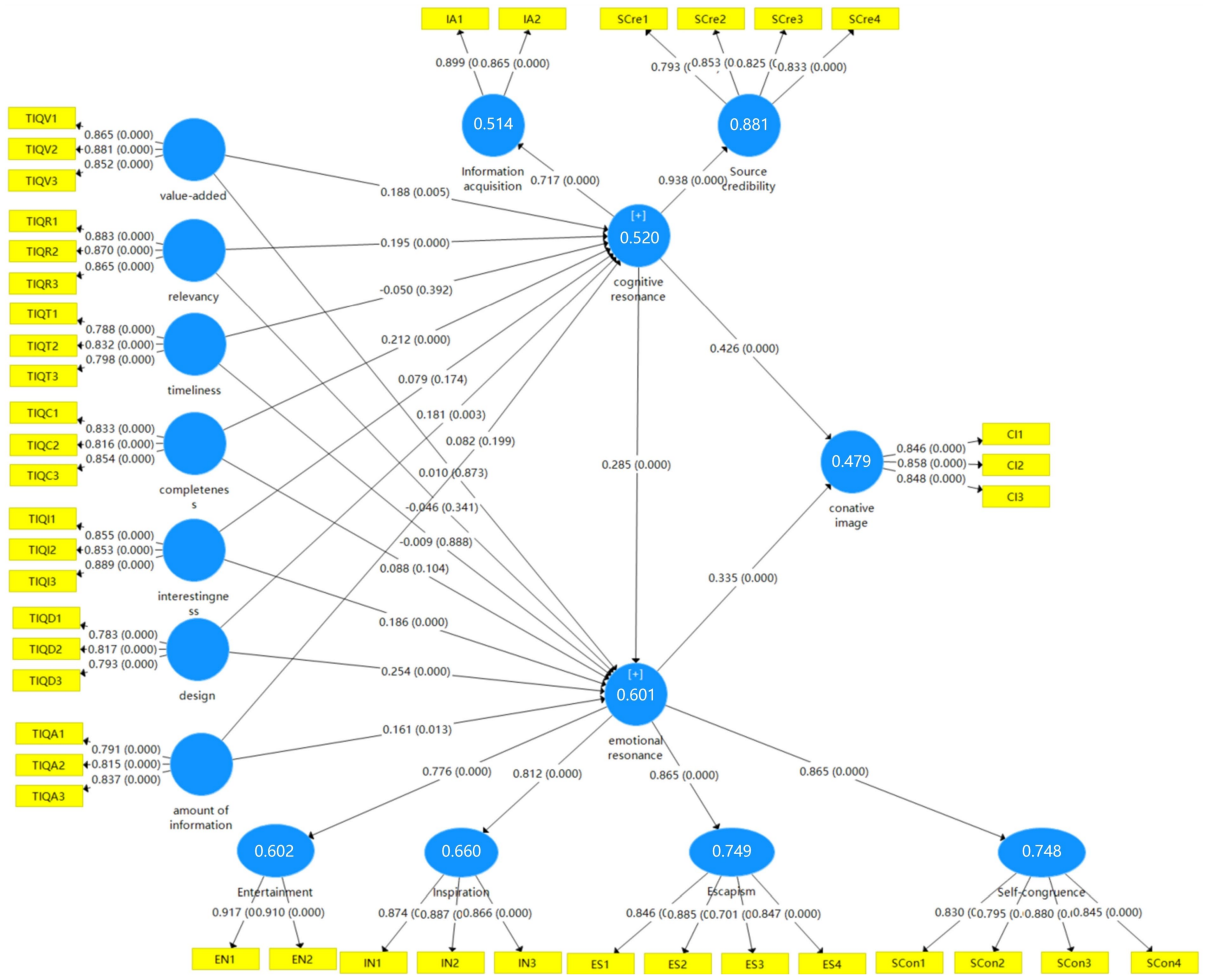
Results of PLS-SEM analysis.

This study used a bootstrapping technique of 5,000 re-samples to examine the significance of the statistical hypothesis model (Hair et al., 2017). The results of PLS-SEM are shown in Figure 2; Table 5. The R-squared values of the three endogenous latent variables (cognitive resonance, emotional resonance, and conative image) were 0.520, 0.601, and 0.479, respectively. Four dimensions of online tourism information quality (value-added, relevancy, completeness, design) significantly influence cognitive resonance (*β*_value-added_ = 0.188, value of *p* = 0.005; *β*_relevancy_ = 0.195, value of *p* = 0.043; *β*_completeness_ = 0.212, value of *p* < .001; *β*_design_ = 0.181, value of *p* = 0.003), supporting hypotheses H1 (a), H1 (b), H1 (d), H1 (f). Three dimensions of online tourism information quality (interestingness, design, amount of information) significantly influence emotional resonance (*β*_interestingness_ = 0.186, value of *p* < 0.001; *β*_design_ = 0.254, value of *p* < 0.001; *β*_amount of information_ = 0.161, value of *p* = 0.012), supporting hypotheses H2 (e), H2 (f), H2 (g). Cognitive resonance significantly influences emotional resonance (*β* = 0.285, value of *p* < 0.001) and conative image (*β* = 0.426, value of *p* < 0.001); emotional resonance significantly influences conative image (*β* = 0.335, value of *p* < 0.001). Thus, the hypotheses of H3 and H4 were supported. To assess whether the omitted constructs have a substantial effect on endogenous constructs, effect size *f*^2^ values are to be calculated (Hair et al., 2017). The values of *f*^2^ for the above significant paths ranged from 0.027 to 0.201, all higher than Cohen’s (2013) criteria effect of size 0.02.

**Table 5:** Results of the hypothetical model of PLS-SEM.

		Path coefficient	*T* statistics	*p* values	*f*-square	Support?
	*R*^2^ value for cognitive resonance = 0.520					
H1	(a) value-added -> cognitive resonance	0.188	2.796	0.005	0.027	yes
	(b) relevancy -> cognitive resonance	0.195	3.413	0.001	0.043	yes
	(c) timeliness -> cognitive resonance	−0.050	0.855	0.392	0.002	no
	(d) completeness -> cognitive resonance	0.212	3.889	0.000	0.042	yes
	(e) interestingness -> cognitive resonance	0.079	1.358	0.174	0.006	no
	(f) design -> cognitive resonance	0.181	3.006	0.003	0.029	yes
	(g) amount of information -> cognitive resonance	0.082	1.298	0.194	0.007	no
	*R*^2^ value for emotional resonance = 0.601					
H2	(a) value-added -> emotional resonance	0.010	0.160	0.873	0.000	no
	(b) relevancy -> emotional resonance	−0.046	0.958	0.338	0.003	no
	(c) timeliness -> emotional resonance	−0.009	0.141	0.888	0.000	no
	(d) completeness -> emotional resonance	0.088	1.646	0.100	0.009	no
	(e) interestingness -> emotional resonance	0.186	3.721	0.000	0.040	yes
	(f) design -> emotional resonance	0.254	5.145	0.000	0.067	yes
	(g) amount of information -> emotional resonance	0.161	2.517	0.012	0.031	yes
H3	cognitive resonance -> emotional resonance	0.285	4.477	0.000	0.097	yes
	*R*^2^ value for conative destination image = 0.479					
H4	(a) cognitive resonance -> conative image	0.426	5.875	0.000	0.201	yes
	(b) emotional resonance -> conative image	0.335	4.862	0.000	0.124	yes

## 6. Discussion

### 6.1. Conclusion

Online travel information quality has a significant effect on cognitive resonance in four dimensions (value-added, relevancy, completeness, and design), and among the four dimensions, completeness has the greatest impact, relevancy ranks second. Three dimensions (interestingness, design, and amount of information) of OTIQ have a significant effect on emotional resonance, and among the three dimensions, design has the greatest impact. This means that complete and relevant information is what audiences are searching for, and design (interface layout and attractive headings) can trigger their emotions. This study also found that cognitive resonance and emotional resonance positively influence conative destination image. These results are consistent with Mohanty et al.’s (2022) study that cognitive resonance and emotional resonance stimulated by the information on Travel Vlogs have an effect on travel intention.

### 6.2. Theoretical implications

Although researchers have classified OTIQ into content cues and non-content cues (Kim et al., 2017; Rodríguez et al., 2019), they did not distinguish how content cues and non-content cues affect audiences’ psychological status. This study indicates that content cues (e.g., relevancy, completeness) strongly stimulate tourists’ cognitive resonance, and non-content cues (e.g., design) strongly stimulate tourists’ emotional resonance. Tourists search for travel information on social media and have a feeling of cognitive resonance once they find the content is relevant and useful for planning the trips, and they have an emotional response once they have an impression of the design of the social media. This study contributes to online tourism marketing research by explaining how online travel information influences tourists’ psychological resonance.

Previous studies have applied the concept of resonance in destination image tourism research (e.g., Cheng et al., 2020), but no studies have evaluated the relationship between cognitive resonance and emotional resonance and no study has compared which resonance has a stronger effect on the formation of the destination image. This study provides evidence to support that cognitive resonance is more important than emotional resonance in forming a conative image of a destination. Cognitive resonance not only directly affects the formation of a conative image but also indirectly affects the formation of a conative image through emotional resonance. This implies that after customers receive online tourism information, they may first filter out information that matches their cognition, thereby affecting emotional resonance. In addition, by applying the concept of resonance, this study provides a complete picture of how OTIQ can create the conative image of a destination. This study contributes to destination image research by extending the application of resonance theory and examining the role of cognitive resonance and emotional resonance in forming a conative destination image.

### 6.3. Practical implications

This study provides marketers with important insights into online tourism marketing. DMOs should carefully design the information posted on social media to effectively market the destination. The information provided should be value-added, relevancy, and completeness. For example, cooperate with influential KOLs to publish the cultural customs of tourist destinations, Instagram-worthy locations, niche attractions, trending stores, travel itineraries, and other information in a thematic manner on social media. In addition, the interface design is also important. For example, a well-designed virtual appearance interface (subcultural beauty, color scheme, layout of images and video modules) can make a destination stand out from the crowd of online information (Jamshidi et al., 2021). These designs can inspire not only cognitive resonance but also direct emotional resonance.

Social media are one of the strongest marketing mediums in the world, so the results of this study are not only applicable to destination marketing but also applicable to other online tourism marketing areas. For example, hotels can push a large number of esthetically pleasing products and brand information related to target customers on social media to attract customers’ attention, while paying attention to customers’ reactions to each product information in order to make further online marketing. Travel agents should use precision marketing to post complete and valuable travel information on social media, design different packages for multiple users, and use attractive interfaces to appeal to customers’ emotional resonance. High-quality online information helps to gain customers’ recognition and earn a corporate reputation.

### 6.4. Limitations and future research

The targets of this study were the tourists before visiting a destination. Further research can compare the differences in the paths of two groups of tourists who have and have not visited the destination before. The data were collected in China. Further research is recommended to collect data in different countries to compare any differences in forming resonance and the conative image of a destination. This research model only consists of OTIQ, resonance, and conative image, researchers can extend this research model with other constructs to understand the impact of QTIQ and resonance on destination image in a comprehensive way.

## Data availability statement

The original contributions presented in the study are included in the article/supplementary material, further inquiries can be directed to the corresponding author.

## Ethics statement

Ethical review and approval was not required for the study on human participants in accordance with the local legislation and institutional requirements. Written informed consent from the participants to participate in this study was not required in accordance with the national legislation and the institutional requirements.

## Author contributions

XuW: idea inception, data collection, data analysis, and writing–original draft. XiW: idea inception and refine, writing–review and editing, evolution research goals, and aims. IL: experiment design, data curation, writing–review and editing, evolution research goals, and aims. All authors contributed to the article and approved the submitted version.

## Conflict of interest

The authors declare that the research was conducted in the absence of any commercial or financial relationships that could be construed as a potential conflict of interest.

## Publisher’s note

All claims expressed in this article are solely those of the authors and do not necessarily represent those of their affiliated organizations, or those of the publisher, the editors and the reviewers. Any product that may be evaluated in this article, or claim that may be made by its manufacturer, is not guaranteed or endorsed by the publisher.
